# Investigating Structural Defects in Extra Hard Cheese Produced from Low-Temperature Centrifugation of Milk

**DOI:** 10.3390/foods12173302

**Published:** 2023-09-02

**Authors:** Luca Bettera, Marcello Alinovi, Paolo D’Incecco, Monica Gatti, Eleonora Carini, Luisa Pellegrino, Elena Bancalari

**Affiliations:** 1 Department of Food and Drug, University of Parma, 43124 Parma, Italy; luca.bettera@unipr.it (L.B.); marcello.alinovi@unipr.it (M.A.); eleonora.carini@unipr.it (E.C.); elena.bancalari@unipr.it (E.B.); 2 Department of Food, Environmental and Nutritional Sciences, University of Milan, 20133 Milan, Italy; paolo.dincecco@unimi.it (P.D.); luisa.pellegrino@unimi.it (L.P.); 3 SITEIA.PARMA Interdepartmental Centre, University of Parma, 43124 Parma, Italy

**Keywords:** hard cheese, cheese defects, cheese ripening, centrifugation, raw milk

## Abstract

The present study investigated some physico-chemical and microbiological traits of 20-month ripened hard cheeses produced from low-temperature high-speed centrifuged raw milk that developed a structural defect consisting of eyes or slits in the paste. Cheeses obtained using the same process and that did not develop the defect were used as controls. The colour, texture, moisture, water activity, proton molecular mobility, microstructure, extent of proteolysis, and viable microorganisms have been evaluated in all the cheese samples, and the significant differences between the defective and non-defective cheeses have been critically discussed. At a microstructural level, the defects caused fat coalescence and an unevenly organised protein matrix with small cracks in the proximity of the openings. The different fat organisation was correlated to a different transverse relaxation time of ^1^H population relaxing at higher times. The textural and colour features were not different from those of the control cheeses and were comparable with those reported in the literature for other long-ripened hard cheeses. On the other hand, the defective cheeses showed a higher moisture level and lower lactobacilli and total mesophilic bacteria concentrations, but the microbial origin of the defect remains an open hypothesis that deserves further investigation.

## 1. Introduction

Visual appearance and texture are important factors to determine the quality and consumer acceptance of cheeses [1,2,3]. The occurrence of openings in the cheese body can have physico-chemical and/or microbiological origin, both related to the milk preparatory treatment and cheese manufacturing conditions. The presence of openings represents a typical feature of certain cheese types, such as the holes in Roquefort caused by unpressed curd or the so-called “eyes” in the Dutch- and Swiss-type cheeses due to gas production by specific bacteria [4]. The size, number, shape, and distribution of the eyes are extremely important quality parameters, especially in Swiss-type cheeses where the eye is mainly the result of propionic acid fermentation involving the conversion of lactate into propionate, acetate, and CO_2_ during warm room storage [5]. On the other hand, holes or eyes represent a defect in blind cheeses, such as the Italian hard cheeses. This cheese variety is characterised by a compact and homogeneous texture without any kind of opening thanks to strong cohesion among the curd granules ensured by the vat cooking as well as the absence of late fermentations during ripening. These conditions favour the formation of crystals and spots [6].

Both early and late blowing are the most common structural defects of microbial origin in hard cheeses. While the first occurs within a few days after production and is caused by coliform bacteria [7], late gas formation arises after a few months as a consequence of lactate and/or citrate fermentation. Late blowing is a problem in hard and semi-hard cheeses since it generally also causes an unpleasant flavour. It is mainly caused by the spore former *Clostridium tyrobutyricum*, but other *Clostridium* species such as *C. sporogenes*, *C. beijerinckii*, and *C. butyricum* were also found to be responsible for the defect [8,9]. The main sources of contamination are thought to be silage in cow feeding and unhygienic animal bedding [10]. Different methods have been proposed to prevent the late blowing defect, such as the addition of nitrate or lysozyme [11], the addition of protective cultures containing lactic acid bacteria strains biologically active against gram-positive bacteria [12,13,14], and the centrifugation (also referred as “bactofugation”) or microfiltration of milk [15].

In previous studies, we demonstrated that low-temperature high-speed (LTHS) centrifugation effectively reduced the number of clostridia spores in raw milk [14], although the treatment caused a parallel selection of the milk microbiota that reflected on the proteolytic pathways of the derived cheeses [15]. Furthermore, a few cheeses produced using this pre-treatment presented openings at the end of the ripening period. Although these structural defects do not necessarily impair the taste and flavour of the cheese, their appearance does not comply with the consumers’ expectations for hard cheese types, which are commonly characterised by a compact and uniform structure. As a consequence, depending on their characteristics, these defective cheeses can undergo different utilisation, such as the preparation of grated, processed, or powdered cheese [16,17,18]. For this reason, it is of great interest to deepen the knowledge about defective cheeses in order to provide useful information for the definition of the process parameters and the prediction of the final product’s features.

The aim of this study was to evaluate some physico-chemical and microbiological traits of hard cheeses that, although they were produced using LTHS centrifuged milk, developed a structural defect in the late-ripening period (20 months). These cheeses were compared with control cheeses that did not develop the defect. This research provides insights into the possible origin of the described cheese defect and represents an integration of the knowledge previously acquired about the characteristics of LTHS centrifuged raw milk [19] and the derived hard cheeses [20].

## 2. Materials and Methods

### 2.1. Cheese Production and Sampling

Raw bulk milk was centrifuged as described in [19] for configuration 1. Briefly, after fat separation by natural creaming at 8–12 °C for 10 h, partly skimmed milk (fat 2.2 g/100 mL) was submitted to single centrifugation using a one-phase centrifuge (CSI-230-01-772, Westfalia, Wiedenbrück, Germany) operating at 39 °C at a flow rate of 21,000 L/h. After centrifugation, the milk was held in a degassing tank at 13 °C for 4 h and then transferred to the cheese vat. Hard cheeses were produced following the technology described in [21].

Briefly, a thermophilic natural whey starter (titratable acidity: 30–32°SH/50 mL), obtained from the residual whey of the previous days’ cheesemaking of the same hard cheese, and calf rennet were added to coagulate the vat milk. No lysozyme was added. The curd was cut into small granules and heated to 52–54 °C. The cheeses were moulded for 48 h to allow lactic acid fermentation and then salted in brine for 18–20 days, leading to a salt content equal to 1.5% at the end of the ripening.

After 20 months of ripening, three cheeses (hereafter shortened to DC, defective cheese) produced in three different days were selected for the study since they developed structural defects verified by X-ray imaging (TDI Packsys, Vernon Hills, IL, USA). Three additional cheeses, produced in the same three days using the same milk but in different vats, without the defect (hereafter shortened to NDC, non-defective cheese) were analysed as controls. A vertical slice (10 cm thick) was sampled from the middle part of the cheese wheel and transported to the laboratory under refrigerated conditions for the analyses.

For the image analysis, both faces of the cheese slice were scanned and data were acquired. The cheese slice was then divided for the following analyses: half of the slice was grated (after removal of 4 cm of rind) for the determination of the moisture content, water activity, low-resolution ^1^H NMR, capillary zone electrophoresis, and viable microbial counts; one quarter was dedicated to confocal laser scanning microscopy; and one quarter was used for colour characterisation and then for texture analysis. For the last two analyses, 9 acquisitions were replicated in the exact positions corresponding to the coordinates of 30, 50, and 70% of the quarter-slice’s height and length.

### 2.2. Image Analysis

Image analysis was carried out to identify and measure the per cent porosity for the DC samples, as the NDC did not present any opening in the cheese paste. Images of the two faces of the cheese slice were acquired using a Hewlett Packard Scanjet 8200 scanner (Palo Alto, CA, USA) with a resolution of 600 dpi (corresponding to 236 pixels cm^−1^) and saved in TIFF format. A black background was used to enhance the contrast of the acquired images, which were processed as described in [22].

To evaluate the opening size distribution, the minimum, maximum, mean, and 25th, 50th, and 75th percentiles (D25, D50 and D75) of the opening area (mm^2^) were recorded. The cheese porosity (%) was calculated according to Equation (1):

(1) $$\text{Porosity}\;\%=\frac{\sum \text{openings}\;\text{areas}\;({mm}^{2})}{\text{cheese}\;\text{section}\;\text{area}\;({mm}^{2})\;}\times 100$$

The openings were differentiated into “eyes” and “cracks/slits” based on the parameter eccentricity (*e*) of the ellipse that has the same second-moments as the region, as calculated by the software using Equation (2):

*e* = *c/a* (2)

where *c* is the distance between the foci of the ellipse, and *a* is its major axis length. The value is between 0 (the ellipse is a circle) and 1 (the ellipse is a line segment); the openings with an eccentricity value lower than 0.9 were classified as “eyes”, and the openings with a value equal to or higher than 0.9 were classified as “cracks”.

### 2.3. Cheese Physico-Chemical Properties

#### 2.3.1. Texture Analysis

An analysis of the cheese paste texture was performed for both the DC and NDC samples by means of a TA.XTplus Texture Analyzer (Stable Micro Systems, Godalming, UK) equipped with a 30 kg load cell and a 3 mm diameter stainless steel cylindrical probe (SMS P/3, Stable Micro Systems). A penetration test was carried out according to the procedure described in [16]. Young’s modulus (MPa), the stress (MPa), and strain (-) at fracture were derived from the true strain (ε) and true stress (δ) parameters, calculated using Equations (3) and (4), respectively [23,24]:

(3) $$\varepsilon =ln(h_{0}/h_{0}-∆h)$$

(4) δ(t)=F(t)/A(t)

where *h*_0_ is the original height of the sample, ∆*h* represents the change in height, *F(t)* is the force at time (t), and *A(t)* is the surface area at time (t).

#### 2.3.2. Colorimetric Characteristics

The colour of the DC and NDC was measured using a Minolta Colorimeter (CM 2600d, Minolta Co., Osaka, Japan) equipped with a standard illuminant D65. CIELAB colour space was considered, with observer = 10° and the target status = SCI/100. The parameters L* (lightness, black = 0, white = 100), a* (redness > 0, greenness < 0), and b* (yellowness > 0, blue < 0) were measured through the whole sample surface in order to obtain a representative value of each parameter for the cheese paste.

#### 2.3.3. Moisture Content and Water Activity

The moisture content of the DC and NDC samples was measured by oven-drying at 102 °C [25] until a constant weight was reached. The data are expressed as the moisture % (*w*/*w*).

The water activity (aw) of the cheese samples was measured at 25 °C using an AquaLab Water Activity Meter Series 3TE with an internal Peltier temperature control device (Decagon Devices, Inc., Pullman, WA, USA). Before the analyses, the instrument was calibrated in the aw range of 0.846–1.000 using saturated salt solutions and distilled water.

#### 2.3.4. Low Resolution ^1^H NMR

An evaluation of the ^1^H molecular mobility and the dynamics of the DC and NDC samples were performed at 25.0 ± 0.1 °C using a low-resolution ^1^H NMR spectrometer (Minispec, Bruker, MA, USA; frequency 20 MHz, magnetic field strength 0.47 T). The cheese paste was sampled using a cork borer and transferred into NMR tubes (outer diameter of 10 mm) that were filled up to a 10 mm height. To avoid moisture loss during the analysis, the tube was sealed with laboratory film. ^1^H T_2_ spin–spin relaxation curves were measured with a Carr–Purcell–Meiboom–Gill (CPMG) pulse sequence by performing 12 scans for each replication, with an RD of 3.5 s (>5 ^1^H T_1_), an interpulse spacing (τ) of 80 μs, and 30,000 data points. The ^1^H T_2_ relaxation curves were analysed as quasi-continuous distributions of the relaxation times using UPENWin software v.1.04 (Alma Mater Studiorum, Bologna, Italy). A multiexponential model was used to quantify the relaxation times and abundance of the detected proton populations using Sigmaplot, v.10 (Systat Software Inc., Palo Alto, CA, USA) as previously reported [26,27].

### 2.4. Confocal Laser Scanning Microscopy

Confocal laser scanning microscopy (CLSM) was applied to study the microstructure of the DC and NDC. Sampling was carried out as previously performed by D’Incecco and co-workers (2020) with some modifications [28]. Three portions of cheese (2 × 2 × 1 mm) were cut using a razor blade from each cheese slice at a 5 cm depth from the rind. Additional sampling was performed close to the defect for the DC. The cheese portions were stained within embryo dishes (Electron Microscopy Sciences, Hatfield, PA, USA) using Nile Red (Sigma Aldrich, St. Louis, MO, USA) to visualise the fat and Fast Green FCF (Sigma Aldrich) to visualise the protein. Just before staining, both stock solutions of Nile Red (1 mg/mL in dimethyl sulfoxide) and Fast Green (1 mg/mL in Millipore MilliQ water) were diluted tenfold in water. The samples were analysed using an inverted confocal laser scanning microscope, A1+ (Nikon, Minato, Japan). The Nile Red was excited at 488 nm and the emission was collected at 520–590 nm. The Fast Green was excited at 638 nm and the emission was collected at 660–740 nm.

### 2.5. Capillary Zone Electrophoresis (CZE)

The extent of proteolysis in the DC and NDC was investigated through capillary zone electrophoresis (CZE), which allows the separation of intact casein fractions as well as major peptides. The conditions described in [28] were adopted. Briefly, 1 g of grated cheese was dissolved in 10 mL of urea-DTT buffer (pH 8.6) at room temperature for 4 h. The solubilised samples were further diluted 1:5 with the same buffer and then filtered using a 0.22 µm membrane filter (Millipore, Burlington, MA, USA) before analysis. The separation was carried out at 45 °C using a Beckman P/ACE System MDQplus equipped with a 50 cm fused silica column (DB-WAX 126-7012, Agilent Technologies, Milan, Italy). The detection was carried out at 214 nm, and the corrected peak areas, calculated as the peak area/migration time, were used to calculate the ratios between the selected peptides and their parent casein fractions [28].

### 2.6. Microbiological Analysis

To evaluate the viable microorganisms in the DC and NDC samples, different culture media were used. In total, 10 g of grated cheese was suspended in 90 mL of 20 gL^−1^ trisodium citrate (pH 7.5) (Sigma Aldrich, St. Louis, MO, USA) and homogenised for 2 min in a blender at 230 rpm (Seward, London, UK). Decimal dilutions were made in quarter-strength Ringer solution (Oxoid, Basingstoke, UK). The samples were plate-cultured in duplicate for the counting of different microbial groups: mesophilic lactobacilli, on acidified MRS agar (Oxoid) (pH = 5.4, reached by acetic acid addition) and incubated under anaerobic conditions with a gas pack (Fisher Scientific, Rodano, Italy) at 30 °C for 48 h; the total mesophilic count, on Milk Plate Agar (Oxoid) under aerobic conditions at 30 °C for 48 h; yeasts and moulds, on yeast extract dextrose chloramphenicol agar (YEDC, Oxoid, Basingstoke, UK) under aerobic conditions at 30 °C for 5 d; propionibacteria (PAB), according to [29] using P2 agar (containing peptone 5 g; beef extract, 3 g; yeast extract, 5 g; sodium lactate, 1 g; agar 15 g L^−1^) under anaerobic conditions at 30 °C for 7 d; coliforms, on Chromocult^®^ Coliform Agar (Merck KGaA, Darmstadt, Germany) under aerobic conditions at 37 °C for 24 h. For enumeration of the spore-forming bacteria, the homogenised samples were heated to 80 °C for 10 min prior to being cultured. The growth media was tryptone soy agar (TSA, Merck KGaA, Darmstadt, Germany), incubated at 30 °C for 72 h. For aerobic spore-forming bacteria, the sample was plated on the surface, while for the anaerobic ones, 1 mL of each dilution was inoculated in the medium using the pour-plate technique and then covered with a thin layer of the same medium in order to obtain a double-layer and then incubated anaerobically into the jars with the AnaeroGen sachet (Oxoid, Basingstoke, UK).

### 2.7. Statistical Analysis

The cheese samples were all analysed in triplicate unless indicated otherwise. The results are reported as the mean ± standard deviation. To test the significant differences between the samples (α = 0.05), a Welch’s *t*-test was used when results showed a heterogeneous variance to the F-test; when the variance was homogeneous, a Student’s *t*-test was applied. Statistical evaluations were performed using the “stats” package in the R environment [30].

## 3. Results and Discussion

### 3.1. Cheese Openings

**Figure 1:**
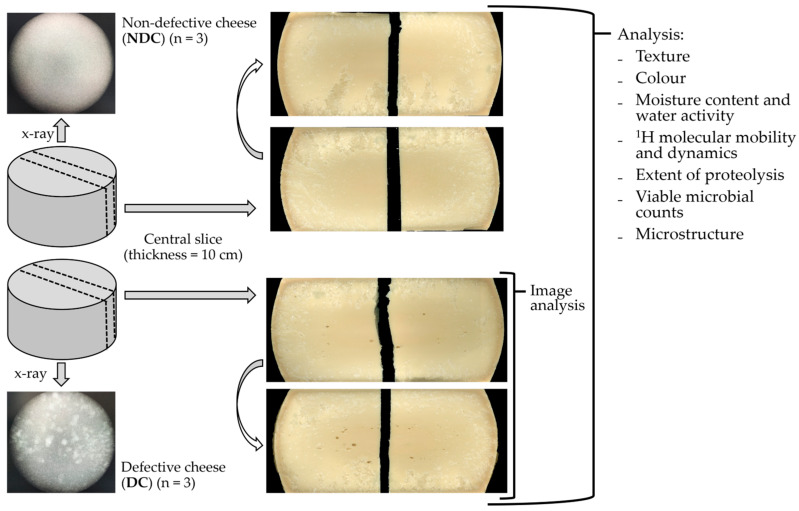
Sampling and analysis schematisation of 20-month ripened non-defective cheese (NDC, n = 3) and defective cheese (DC, n = 3) produced from high-speed centrifuged raw milk.

The appearance of the opening defect is shown in Figure 1. The results of the image analysis conducted to characterise the cheese structure defect are reported in Table 1. The openings caused a porosity of 0.26 ± 0.10% in the DC samples. The largest hole measured, on average, 36.45 ± 1.10 mm^2^ between the replicates, while the smallest measured 0.39 ± 0.03 mm^2^. The mean size was 3.48 ± 1.83 mm^2^, while the D50 was equal to 1.31 ± 0.25 mm^2^, indicating that the opening size follows a right-skewed distribution. Around 75% of the openings were oval- or round-shaped (*e* < 0.9) and, thus, were identified as eyes, while the remaining 25% were cracks/slits (*e* ≥ 0.9). The cheese porosity (0.26 ± 0.10%) was, in general, lower than that reported by other authors. Bettera and co-workers (2020) characterised Nostrano Valtrompia, a PDO [protected designation of origin; [31] hard cheese made with raw milk, and found a porosity ranging between 0.0% and 10.6%, with a mean value of 2.0 ± 2.5%. Instead, Innocente and Corradini (1998) reported a porosity ranging between 3.1% and 18.3% for Montasio PDO cheese [32]. On the contrary, similar porosity values (around 0.2%) were observed in Pecorino cheese [33].

### 3.2. Cheese Physico-Chemical Properties

#### 3.2.1. Cheese Colour and Texture

**Figure 2:**
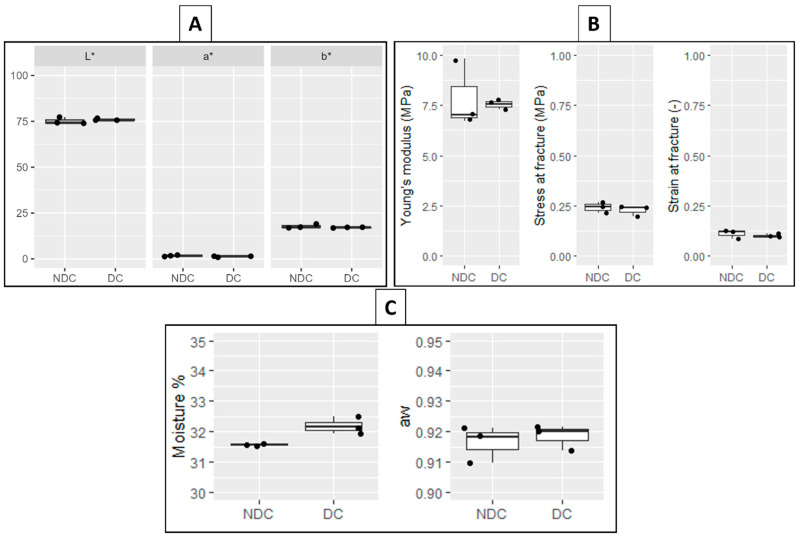
Physico-chemical characteristics of 20-month ripened non-defective cheese (NDC, n = 3) and defective cheese (DC, n = 3): Colour (**A**), texture (**B**), moisture (%), and water activity (*a_w_*) (**C**).

No significant differences were detected in the texture and colour between the DC and NDC (Figure 2A,B). The lightness (L*) of the two cheese types was around 75.5, while the values of the a* and b* parameters were around 1.5 and 17.5, respectively. These values were consistent with those reported for other long-ripened hard cheeses, such as Parmigiano Reggiano PDO [34,35], Montasio [36], Nostrano Valtrompia PDO [22], and Asiago PDO [37,38]. The texture parameters of the stress and strain at fracture were also comparable with those of hard cheeses ripened for 18 [39] or 16 months [22], although our cheeses were harder, showing higher values of Young’s modulus, i.e., 7.87 ± 1.72 MPa and 7.55 ± 0.28 MPa for NDC and DC, respectively.

#### 3.2.2. Cheese Moisture and Water Activity

The values of the moisture (%) and a_w_ of the DC and NDC are reported in Figure 2C. The moisture content was higher in the DC compared to the NDC (32.19 ± 0.28% and 31.57 ± 0.3%, respectively), with the difference being close to the limit of significance (*p* = 0.059). The values of aw were similar between the two samples, with an average value of 0.918. Both the moisture and the aw values were consistent with those found for other hard cheeses with a similar ripening time [28,40].

### 3.3. Cheese Microstructure

**Figure 3:**
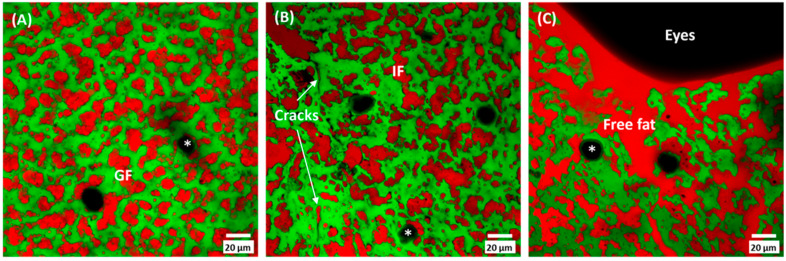
Confocal laser scanning microscopy of non-defective (**A**) and defective (**B**,**C**) cheeses. Fat was organised as globular fat (GF) in non-defective cheese, while irregular fat pools (IF) were visible in defective cheese. Cracks of the protein matrix were observed in defective cheese only. Asterisks (*) indicate calcium phosphate crystals. Bar is 20 µm in length.

The microstructure of cheeses was investigated by confocal laser scanning microscopy (CLSM, Figure 3). Differences were observed between the DC and NDC involving both the fat and protein components. The fat was mostly organised as irregular globular-shaped areas of partially coalesced fat globules within a continuous protein network in the NDC. This organisation is in accordance with previous observations of cheese microstructure in long-ripened hard cheese [28]. In contrast, the fat fully coalesced in the DC, and the fat matrix was shown to be damaged and very irregular. Large pools of free fat formed in the proximity of the defect, especially at the inner surface of the eye (Figure 3C). The protein matrix was unevenly organised with small cracks of a few microns in size that were frequently filled by free fat. In cheeses with an inelastic structure, like those considered in the present study, the high overpressure of the produced CO_2_ leads to the spontaneous formation of cracks in the weaker zones of the cheese [5]. The microstructure of the DC in the areas not affected by defects was comparable to that of the NDC. All the cheeses were characterised by the presence of unstained spots (asterisks in Figure 3) corresponding to calcium phosphate crystals, as previously identified by micro-Raman in the same type of cheese [41]. These crystals formed as an effect of the super-saturation of calcium phosphate salt, which increases during cheese ripening, and were confirmed to be widespread throughout the whole cheese.

### 3.4. Cheese Proteolysis

The extent of primary proteolysis was evaluated by CZE, which separated intact caseins as well as major casein fragments cleaved by chymosin or indigenous milk enzymes. The ratios between the peak areas of the major fragments and the respective parent caseins were calculated. Specifically, the ratios αs_1_-I/αs_1_, αs(f1–23)/αs_(1+0)_, αs_1_-PL/αs_1_-CN, and ɣ/β were considered (Table 2). No significant differences were found between the ratio values in the DC and NDC, suggesting an equivalent enzymatic activity occurs in the two cheese types. Both αs_1_-I, corresponding to f(24–199), and αs(f1–23) fragments result from a side activity of chymosin towards αs_1_-CN. A progressive decrease in the αs_1_-I/αs_1_-CN ratio occurs during ripening as an effect of the faster degradation of αs_1_-I-CN than αs_1_-CN [28]. In contrast, β-casein is known to be primarily cleaved by plasmin, especially in cooked cheeses [42]. Since ɣ-caseins are quite stable, the ɣ-casein/β-casein ratio increases during ripening [43]. The activity of plasmin is also exerted towards the αs_1_-casein. However, even the αs_1_-PL/αs_1_-casein ratio did not show differences between the DC and NDC. It is known that proteolysis is very important for the development of a defined texture and flavour in ripened cheeses. In fact, different proteolysis pathways may stimulate the growth of some microbial populations instead of others, depending on the availability of different patterns of the small peptides and free amino acids used as a growth substrate [44]. However, this would not be the case due to the comparable casein degradation observed in the DC and NDC.

### 3.5. Low-Resolution ^1^H NMR Analyses

**Figure 4:**
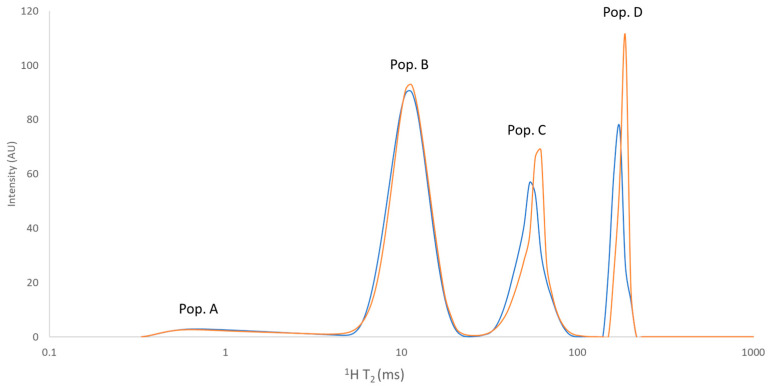
Representative ^1^H T_2_ relaxation distribution of populations measured by CPMG sequence of DC (orange line) and NDC (blue line) samples.

The results of the low-resolution ^1^H T_2_ NMR analyses (Table 3) showed the presence of four ^1^H populations, named from the least to the most mobile as A, B, C, and D. As can be observed from the representative ^1^H T_2_ distributions of the relaxation times for the DC and NDC (Figure 4), the four populations were quite well resolved. Four proton populations relaxing in similar time ranges were previously found in long-ripened hard cheeses [45,46].

In particular, population A (T_2_ around 1 ms) has been previously attributed to the protons of water molecules tightly bound to the macromolecules or the protons belonging to the most rigid casein portions (intra-micellar water fraction), and population B (T_2_ around 10 ms) has been attributed to water in exchange with the casein surface (inter-micellar water fraction) or the protons belonging to the most flexible regions of casein [45,46]. Bordoni and co-workers (2011) attributed populations C (T_2_ around 50 ms) and D (T_2_ around 170 ms) to the solid and liquid lipid fractions, respectively [45]; the same attribution was also performed by De Angelis Curtis and co-workers (2000) [46]. However, it is difficult to support the attribution of population C to solid fat, as the value of T_2_ for protons in the solid fat phase (that can be expected in the order of μs) cannot be observed on the timescale of CPMG experiments [47,48,49]. Mulas and co-workers (2016), who studied Grana Padano PDO cheese by performing MRI analyses, attributed population D to both a proton fraction of water molecules exhibiting less interaction within the protein matrix and to a fraction of liquid fat [50].

Significant differences (*p* < 0.05) between the NDC and DC samples were observed in the case of T_2D_ and T_2B_: the NDC showed shorter transverse relaxation times of population B and population D compared to the DC samples. In particular, the different organisation of the fat domain observed through the CLSM analyses can be related to the different transverse relaxation time of population D (170 ms for NDC vs. 180 ms for DC). An increase in T_2D_ may indicate greater conformational freedom of the fat protons, which can be the consequence of a coarser and more irregular distribution of fat globules in the matrix of the DC rather than the NDC [51] and the presence of free fat in the proximity of the defects. The lower T_2B_ reported in the case of the NDC compared to the DC may also be indicative of a different structural organisation of the protein matrix, characterised by a higher capacity for reducing the molecular mobility of the interstitial water fraction.

No significant differences between the NDC and DC samples were observed in the relative abundance (area %) of the four ^1^H populations. Population B was the most abundant, representing around 55%. Overall, our relative abundance values were in good agreement with those found by De Angelis Curtis and co-workers (2000) in the same type of cheese [45].

### 3.6. Microbiological Analyses

The concentrations of viable microorganisms found in the 20-month ripened cheeses are reported in Table 4. Coliforms and spore-forming bacteria (both aerobic and anaerobic) were absent in the DC and NDC. Except for yeasts/moulds, the DC had a more variable microbiological profile between the replicates, as shown by the wider boxplots. The DC and NDC had a similar average concentration of yeasts/moulds and PAB; the latter was the microbial group that showed the highest variability among the replicates. Differences were instead noticed in the lactobacilli and total mesophilic counts. The latter was significantly higher in the NDC (log CFU/mL = 4.86 ± 0.14, against the average value of 3.87 ± 0.41 of the DC). The NDC also showed a higher concentration of lactobacilli, with a *p*-value = 0.063, which was slightly above the significance level (α = 0.05).

D’Incecco and co-workers discussed the influence of LTHS centrifugation in modifying the LAB composition in milk [19]. They observed a selective effect of the centrifugation that reduced rod-shaped LAB preferentially. This was later confirmed from an analysis of ripened cheeses produced with centrifuged milk: these had lower viable lactobacilli and *Lacticaseibacillus* spp. concentrations compared to the control cheeses made from non-centrifuged milk [20]. With respect to the DC analysed in this study, the selective removal may have unbalanced the LAB species abundance in the raw milk, leading to a different interaction between the PAB and LAB, possibly resulting in the stimulation of propionic acid fermentation. This phenomenon was already demonstrated to occur during cheese ripening by Fröhlich-Wyder and co-workers (2002) [43]. The authors found that the absence of facultatively heterofermentative lactobacilli made PAB able to produce excessive CO_2_ with the consequent formation of larger eyes. This finding could explain our results where the DC with eyes was associated with lower counts of lactobacilli. Facultatively heterofermentative non-starter LAB, such as *Lacticaseibacillus casei* and *Lacticaseibacillus rhamnosus*, are, in fact, known to slow down propionic acid fermentation, while thermophilic starter species, such as *Lactobacillus helveticus*, may stimulate late fermentation, representing a risk of structural defects of eyes in the ripened cheese or slits in the case of a less elastic cheese paste. The interaction between LAB and PAB is known to be crucial to achieving and controlling the structural quality of hard cheeses, including the control of CO_2_ production [52]. In the present study, including replicates from three cheesemaking events carried out on three different days, the PAB concentration in the 20-month ripened DF cheeses was highly variable. Although the significance of the difference did not confirm what was previously observed, the microbiological origin of the defect remains open as a possible cause [43]. Thus, this hypothesis deserves to be further investigated by following the process throughout the long cheese ripening time and analysing cheese samples at different times in order to evaluate the vital microbial cells also through the level of gene expression of different metabolites.

The development of structural defects in cheese, in the form of eyes or openings, can also be guided by non-microbiological factors, especially when the raw milk undergoes treatments like centrifugation or microfiltration that, somehow, can modify the native structure of components such as fat globules [5]. Auer and co-workers (2021) and O’Sullivan and co-workers (2016) mentioned that the entrapment of air during moulding of the curd could be responsible for the formation of eye defects [53,54]. In the case of DC, we can speculate that milk centrifugation causes the solubilisation of air that incidentally may be not effectively removed during the subsequent degassing phase. This would explain why the studied defect appeared only seldom.

## 4. Conclusions

Long-ripened hard cheeses are characterised by a compact paste structure. Due to many factors related to the production technology, the cheese can develop structural defects of physico-chemical and/or microbial origin. An improper structure appearance leads to the downgrading of the cheese, even if the taste is not necessarily affected. Late blowing is a well-known defect of hard cheeses that can be avoided using milk centrifugation. Despite the use of low-temperature high-speed (LTHS) centrifugation, we experienced the incidental production of cheeses with a structural defect apparently not directly or solely of microbial origin.

The present study investigated some physico-chemical and microbiological properties of 20-month ripened hard cheeses produced from LTHS-centrifuged raw milk that developed structural defects of eyes/slits opening in the paste. From a mesoscopic point of view, the textural and colour features were not different from the control cheeses produced using the same technology but without defects, and the values were comparable with those of other long-ripened hard cheeses found in the literature. Conversely, at a microstructural level, hard cheeses produced from LTHS-centrifuged raw milk exhibited fat coalescence and unevenly organised protein with small cracks in the proximity of the openings. The different fat organisation was related to a higher mobility of the more mobile proton population detected in the ^1^H T_2_ time frame window and tentatively associated with the more mobile lipid domain.

The defective cheese showed a higher moisture content, while the proteolysis progress was not significantly different. These cheeses had a lower, although not statistically different, lactobacilli concentration that could have stimulated the propionic pathway by PAB and, as a consequence, the defect formation. These results not only offer new insight into the description and consequent prevention of hard cheese defects but also demonstrate that several cheese properties were not altered by the defect, providing useful information about the possible use of defective cheeses. However, further experiments are necessary to better understand the microbiological and physico-chemical phenomena that can explain the origin of these defects during the development of the cheese structure.

## Figures and Tables

**Table 1:** Size frequency distribution percentiles (D25, D50, D75), mean, maximum, and minimum size of the defective cheese openings measured using image analysis.

Parameter		Defective Cheese (n = 3)	
		Mean	St. Dev.
Opening area (mm^2^)	Min.	0.39	0.03
	D25	0.71	0.13
	D50	1.31	0.25
	D75	3.48	1.83
	Max.	36.45	12.37
	Mean	3.92	1.10
Porosity (%)		0.26	0.10

**Table 2:** Casein fraction ratios in defective (DC, n = 3) and non-defective (NDC, n = 3) cheeses at 20 months of ripening.

Cheese	αs_1_-I/αs_1_	αs(f1–23)I/αs_(1+0)_	αs_1_-PL/αs_1_	ɣ/β
NDC	0.38 ± 0.04	0.19 ± 0.05	0.68 ± 0.20	4.38 ± 1.45
DC	0.29 ± 0.12	0.13 ± 0.01	0.56 ± 0.06	3.55 ± 0.58
Sign.	n.s.	n.s.	n.s.	n.s.

The results are expressed as the mean ± standard deviation. n.s. = not significant (*p* > 0.05).

**Table 3:** Relative abundance (%) and ^1^H T_2_ relaxation time (ms) of the four proton populations (pop. A, B, C, D) measured by low-resolution NMR relaxometry for non-defective cheeses (NDC, n = 3) and defective cheeses (DC, n = 3).

Cheese	Pop. A (%)	Pop. B (%)	Pop. C (%)	Pop. D (%)	T_2A_ (ms)	T_2B_ (ms)	T_2C_ (ms)	T_2D_ (ms)
NDC	5.44 ± <0.01	55.06 ± 0.08	23.58 ± 0.05	15.92 ± 0.04	1.55 ± 0.12	10.78 ± 0.05	51.10 ± 1.37	170.79 ± 5.68
DC	5.32 ± 0.08	54.55 ± 1.01	23.71 ± 0.79	16.42 ± 0.48	1.62 ± 0.19	11.13 ± 0.19	54.07 ± 2.16	180.54 ± 1.79
Sign. (* *p* < 0.05)	n.s. (*p* = 0.065)	n.s.	n.s.	n.s.	n.s.	*	n.s.	*

The results are expressed as the mean ± standard deviation. n.s. = not significant (*p* > 0.05); * = *p* < 0.05.

**Table 4:** Concentration (log CFU/mL) of the microbial population found in 20-month ripened non-defective cheese (NDC, n = 3) and defective cheese (DC, n = 3).

Cheese	Lactobacilli	Total Mesophilic	Yeasts & Moulds	Propionibacteria
NDC	4.36 ± 0.3	4.86 ± 0.14	3.8 ± 0.73	4.19 ± 0.33
DC	3.52 ± 0.48	3.87 ± 0.41	4.1 ± 0.1	4 ± 0.97
Sign. (* *p* < 0.05)	n.s (*p* = 0.063)	*	n.s	n.s

The results are expressed as the mean ± standard deviation. n.s. = not significant (*p* > 0.05); * = *p* < 0.05.

## Data Availability

The data used to support the findings of this study can be made available by the corresponding author upon request.
